# Role of Vitamin C Supplementation in the Prevention of Premature Rupture of Membranes (PROM) and Preterm PROM: A Systematic Review and Meta-Analysis

**DOI:** 10.7759/cureus.62445

**Published:** 2024-06-15

**Authors:** Banashree Nath, Harsha Gaikwad, Hirok Roy, Sayanti Paul, Vaibhav Kanti

**Affiliations:** 1 Obstetrics and Gynaecology, All India Institute of Medical Sciences, Raebareli, Raebareli, IND; 2 Obstetrics and Gynaecology, Vardhman Mahavir Medical College and Safdarjung Hospital, New Delhi, IND; 3 Anaesthesiology, All India Institute of Medical Sciences, New Delhi, IND; 4 Obstetrics and Gynaecology, All India Institute of Medical Sciences, Kalyani, Kalyani, IND

**Keywords:** micronutrient supplementation, randomised controlled trials, pregnancy, ascorbic acid, rupture of membranes

## Abstract

Vitamin C is a micronutrient assumed to have effects on the occurrence of “preterm premature rupture of membranes” (PPROM) and “premature rupture of membranes” (PROM). The objective of this review was to find the pooled incidence of PROM and/or PPROM between subgroups in relation to dose, mode of therapy (monotherapy vs. combination therapy) and history of PROM/PPROM in previous pregnancies. A search was conducted in the electronic databases (PubMed, Google Scholar, Scopus) from inception to November 2022, using the search terms “Vitamin C”, “Ascorbic acid”, “preterm premature rupture of membrane” and “premature rupture of membrane”. The lists of references of all the selected eligible articles were also searched to find studies of interest. A total of nine randomized controlled trials (published in English) with 16,076 participants involving the supplementation of vitamin C during pregnancy were picked up for analysis. Data management was done using the Review Manager (RevMan 5.3). A statistical test for publication bias was done in jamovi, version 2.3.18. In comparison to placebo, vitamin C supplementation was not found to be significantly effective in preventing the occurrence of PPROM/PROM. However, a low dose of vitamin C and the monotherapy mode of administration significantly decreased the occurrence of PPROM/PROM. Vitamin C has significant beneficial effects in women with a history of PROM in a previous pregnancy. Hence, we conclude that vitamin C administered as monotherapy in low doses (preferably 100 mg/day) has definite benefits in preventing the occurrence of PROM/PPROM with greater advantages seen in those with a history of similar complications in a previous pregnancy.

## Introduction and background

Prelabour or premature rupture of membranes (PROM) is the rupture of membranes before the onset of uterine contractions. When the rupture of membranes occurs both before the onset of labour and at 37 weeks of gestation, it is called preterm PROM (PPROM). The occurrence of PROM is observed in 5% to 10% of all deliveries while PPROM complicates approximately 3% of all pregnancies. Preterm labour is initiated by a rise in local cytokines, disruption in the interaction between matrix metalloproteinases and tissue inhibitors of matrix metalloproteinases, and the rising activity of collagenase and protease along with other factors that may raise the intrauterine pressure [1]. Subsequently, membranes are stretched releasing mediators that activate destructive enzymes in the matrix and the rupture of fetal membranes. Reactive oxygen species (ROS) are generated in the process, acting as a mediator of membrane destruction. Micronutrients are assumed to neutralise the ROS, and hence, the deficiency of nutrients can disrupt the integrity of membranes. Vitamin C is one such micronutrient that reduces oxidative stress by scavenging several ROS [2]. It is a water-soluble antioxidant that cannot be synthesized by the human body. Hence, the quantity and quality of a woman's diet during pregnancy, comprising both micronutrients and macronutrients, have immense effects on fetal growth and development apart from other fetomaternal health outcomes [3]. The blood levels of vitamin C or the dietary intake of vitamin C during pregnancy is a factor assumed to have effects on the occurrence of PPROM/PROM. Siega-Riz et al. [4] in their prospective cohort study found that women with an intake of vitamin C below the 10th percentile before conception had twice the odds of developing PROM. However, in a Cochrane review and meta-analysis, which included all randomized or quasi-randomized controlled trials and examined the effect of vitamin C supplementation in antenatal women, no difference in the risk of PROM (average RR 1.26, 95% CI 0.62 to 2.56; 2674 participants; three studies; I^2^ = 87%), or PPROM (average RR 0.98, 95% CI 0.70 to 1.36; 16,825 participants; 10 studies; I^2^ = 70%; low quality evidence) was observed [5]. However, there are innumerable aspects of vitamin C administration that need to be considered while coming to a conclusion regarding its effects. To the best of our knowledge, there is a lack of a comprehensive analysis of all the factors likely to influence the effects of vitamin C on the occurrence of PROM/PPROM. The objective of this article is to present a comprehensive review of the literature regarding the influence of vitamin C supplementation on the occurrence of PROM/PPROM affecting a multitude of maternal and fetal health conditions and outcomes.

## Review

Methodology

Scope

In this review, we tried to evaluate the effect of vitamin C administration on the chances of premature rupture of membranes. To address this, we framed the following research questions: (1) Is there significant difference in the occurrence of PROM/PPROM following the administration of vitamin C? (2) Is there any significant difference in the occurrence of PROM/PPROM following the administration of high-dose and low-dose (≤250 mg/day) vitamin C? (3) Is there any significant difference in the occurrence of PROM/PPROM following the administration of vitamin C as monotherapy or combination therapy (vitamin E, iron and folic acid)? (4) Does vitamin C administration have a significant role in preventing the occurrence of PROM/PPROM in women with a history of such event in their previous pregnancy?

Search Strategy

Two authors were independently involved in the selection of studies. The studies were identified after a search was conducted in the electronic databases (PubMed, Google Scholar, Scopus) from inception to November 2022. There were no restrictions for the time of publication. The search was concluded on November 30, 2022. The search terms used included “Vitamin C”, “Ascorbic acid”, “preterm premature rupture of membrane” and “premature rupture of membrane”. The search was further refined by the use of appropriate medical subject headings and Boolean operators. The search in the electronic database and assessment of the eligibility of the studies were independently done by two of the authors (BN, SP). All the full texts of the recruited articles were retrieved for further screening. Furthermore, the lists of references of all the selected eligible articles were searched to find any studies of interest missed by the electronic searches. Any discrepancy regarding selection, if arose, was resolved by a third author (VK). Extraction of data was done independently by two investigators (BN, SP). Gross information from each eligible study was extracted without modification and was charted on a table (Table 1) to identify the major themes arising out of studies' results. Differences arising, if any, were settled by discussion and consensus.

**Table 1:** Summary of the effects of micronutrient supplementation on outcomes related to PPROM and/or PROM RCT: randomized controlled trial; NS: not specified; PROM: premature rupture of membranes; PPROM: preterm PROM

S. no.	Author, year	Study design	Study group (SG)	Control group (CG)	Status of SG and CG	Timing of initiation	Risk factors	Intervention (per day supplementation)	Outcome parameters
1	Casanueva et al., 2005 [6]	Double-blind RCT	60	60	Singleton pregnancy	<20 weeks of gestation	NS	Vitamin C, 100 mg vs. placebo	Significantly lower incidence of PROM in the study group
2	Ghomian et al., 2013 [7]	RCT	85	85	Singleton pregnancy	14 weeks’ gestation	PPROM in a previous pregnancy	Vitamin C, 100 mg vs. placebo	Significantly lower incidence of PPROM in the study group
3	Roberts et al., 2010 [8]	Double-blind RCT	4992	4976	Singleton pregnancy	9 to 16 weeks’ gestation	NS	Vitamin C, 1000 mg; vitamin E, 400 IU vs. placebo	No significant difference in the incidence of PPROM between the groups
4	Spinnato et al., 2008 [9]	Double-blind RCT	371	368	Antenatal women seeking prenatal care	120/7 to 196/7 weeks’ gestation	Nonproteinuric chronic hypertension or preeclampsia in the most recent pregnancy	Vitamin C, 1000 mg; vitamin E, 400 IU vs. placebo	Significantly higher incidence of PROM/PPROM in the study group
5	Xu et al., 2010 [10]	Double-blind RCT	1167	1196	Antenatal women having both singleton and multiple pregnancies	12 to 18 weeks’ gestation	NS	Vitamin C, 1000 mg; vitamin E, 400 IU vs. placebo	Significantly higher incidence of PROM/PPROM in the study group
6	Zamani et al., 2013 [11]	Double-blind RCT	32	30	Antenatal women	At 18 weeks’ gestation	PROM and PPROM in a previous pregnancy	Vitamin C, 500 mg vs. placebo	Lower incidence of PPROM in the study group
7	Rumbold et al., 2006 [12]	RCT	935	942	Nulliparous women with singleton pregnancy	14 to 22 weeks’ gestation	NS	Vitamin C, 1000 mg; vitamin E, 400 IU vs. placebo	No significant difference in the incidence of PPROM between the groups
8	Ochoa-Brust et al. [13]	Single-blind RCT	55	55	Antenatal women	12 weeks’ gestation	NS	Vitamin C, 100 mg; ferrous sulphate, 200 mg; folic acid, 5 mg vs. ferrous sulphate, 200 mg; folic acid, 5 mg	No incidence of PROM in either group
9	Kiondo et al., 2014 [14]	RCT	415	418	Antenatal women	12 to 22 weeks’ gestation	NS	Vitamin C, 1000 mg vs. placebo	No significant difference in the incidence of PPROM between the groups

Study Selection

Inclusion criterion: All randomized controlled trials (RCTs) involving the supplementation of vitamin C during pregnancy were included in the systematic review and meta-analysis. Studies that administered the supplementation of vitamin C either alone or in combination with vitamin E, iron and folic acid preparation, and compared this with placebo or iron and folic acid preparation were included in the analysis. Interventions involving a population with either singleton or multiple pregnancies irrespective of parity were included. Any study analysing the incidence of PROM and/or PPROM as a primary or secondary objective in women with or without risk factors was included.

Exclusion criteria: Studies where vitamin C was given as the primary supplement but outcomes other than PROM/PPROM were analysed were excluded. Even studies evaluating the incidence of PROM/PPROM in relation to serum vitamin C levels were not included in the review. Articles published in languages other than English were also excluded.

Data Extraction and Quality Assessment

Each eligible study was used for data extraction, which included bibliographic information (author, publication year), characteristics of antenatal women, reported weeks of gestation for supplementation of vitamin C, inclusion of women with any risk factors, dose and duration of supplementation, information whether vitamin was given as monotherapy or in combination with vitamin E, iron and folic acid, and reported outcomes regarding the incidence of PROM/PPROM. Data was also extracted from each study for the assessment of risk of bias (generation of random sequence, allocation concealment, blinding of participants and personnel, incomplete outcome data, selective reporting) using the Cochrane tool for risk of bias assessment. Two authors (BN, SP) independently assessed the risk of bias. Any disagreements were resolved by a discussion with a third reviewer (HSG).

Primary Outcomes

The correct estimate of the effect magnitude of the occurrence of PROM and/or PPROM in pregnant women supplemented with vitamin C starting in the second trimester was the primary outcome parameter for our systematic review and meta-analysis. We planned to determine the pooled incidence of PROM and/or PPROM between subgroups in relation to dose, mode of therapy (monotherapy vs. combination therapy) and history of PROM/PPROM in a previous pregnancy.

Data Management

The data management was done using the Review Manager (RevMan 5.3; Nordic Cochrane Centre, Cochrane Collaboration, Copenhagen). The data analysis was conducted keeping in mind the research questions framed. For the outcome measures where the comparison was of the number of events of interest occurring between two groups, the variables included ‘number of events of interest occurring’ and ‘total number of events taking place’. The Forest plots generated were evaluated and if there was statistically significant heterogeneity (I^2^ >50%), then the random-effects model was used. RevMan was also used to generate the funnel plots from the recruited studies to identify any publication bias. RevMan was also used to identify the risk of bias of all the recruited studies using appropriate items. A statistical test for publication bias was done in jamovi, version 2.3.18 (jamovi, Sydney).

Results

Literature Search and Study Selection

**Figure 1:**
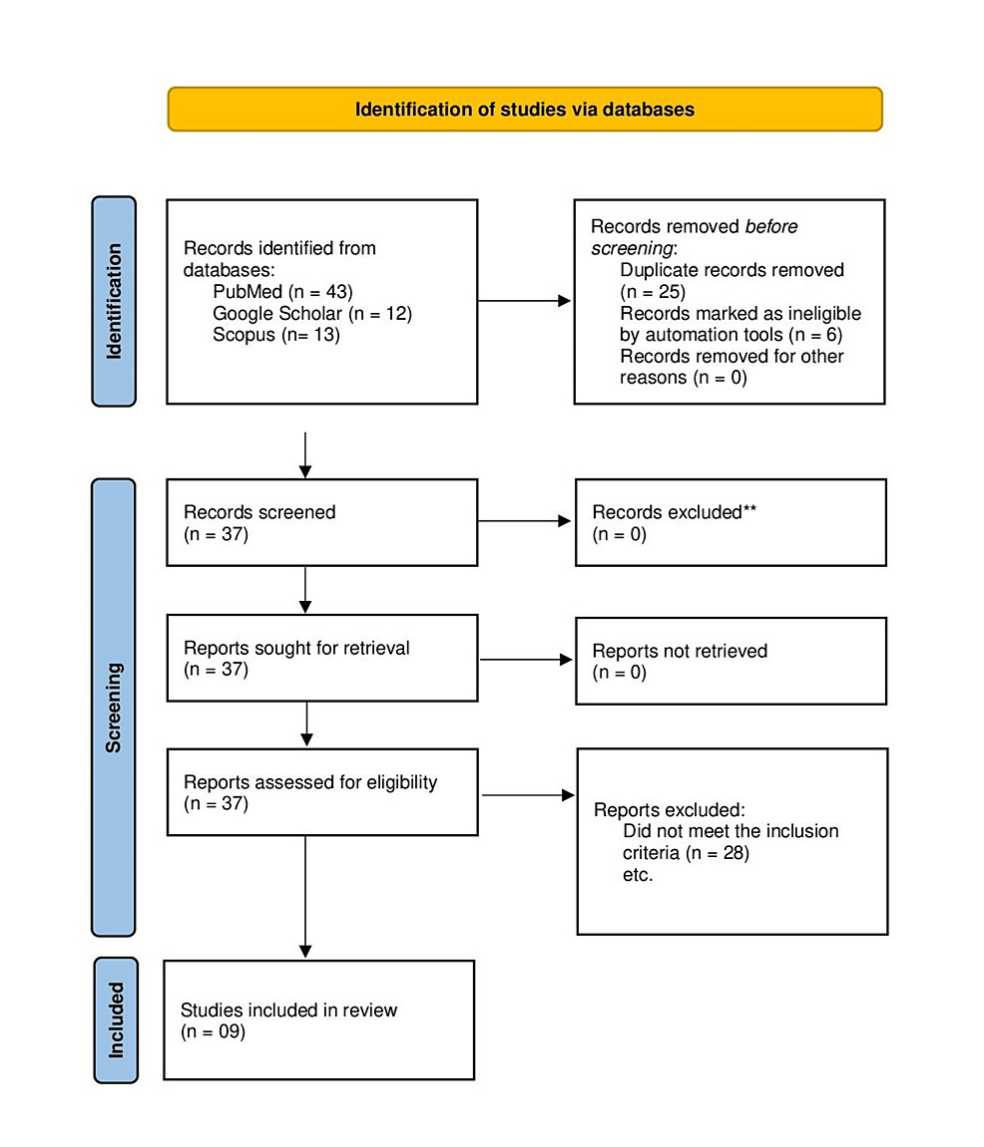
PRISMA flow diagram of search for vitamin C supplementation in the prevention of PPROM/PROM PRISMA: Preferred Reporting Items for Systematic Reviews and Meta-Analyses; PROM: premature rupture of membranes; PPROM: preterm PROM **All 37 studies fulfilled our inclusion and exclusion criteria.

A total of 68 studies were identified using the search strategy mentioned in the Methodology section. Duplicate records were separated and ineligible studies were removed by automation tools. A total of 37 studies were retrieved and assessed for eligibility out of which nine studies (all in English) were finally picked up for further analysis [6-14]. The PRISMA flowchart is presented in Figure 1.

Risk of Bias

**Figure 2:**
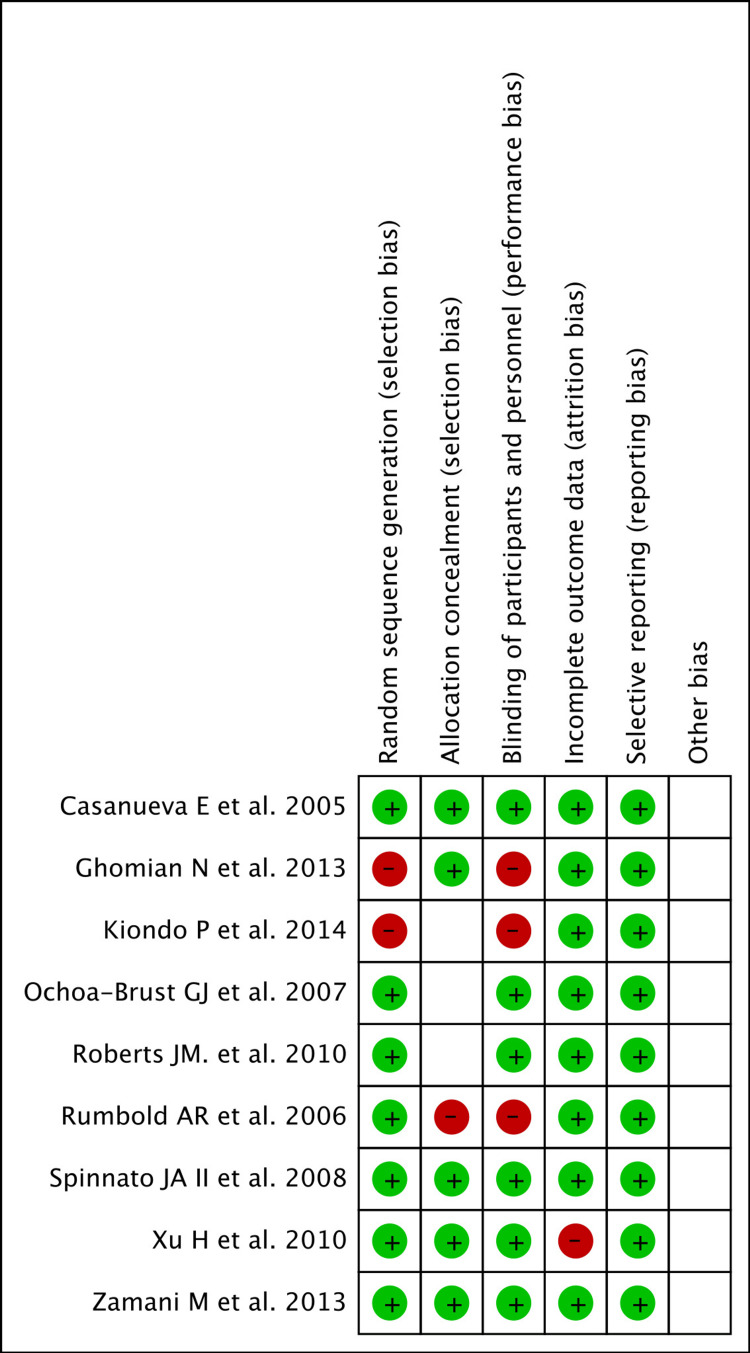
Risk of bias summary: authors' judgements about each risk of bias item for each included study Refer to [6-14]

The Cochrane tool for assessing risk of bias was employed in the present study [15]. The occurrence of PPROM/PROM was explicit which was mostly diagnosed by standardized clinical tests in most of the trials. Because of the conspicuous nature of the observation we were dealing with, blinding of outcome assessment in the trials seemed needless. Hence, the risk for six sections of bias (after the exclusion of blinding of outcome assessment) were assessed as low (green), high (red) and unclear (no colour). Poorer scores were obtained for allocation of concealment in four studies (unclear in most of them) followed by blinding of participants and personnel in three studies. The assessment of random sequence generation (selection bias), incomplete outcome data (attrition bias), and selective outcome reporting (reporting bias) appeared to be adequately present in most studies (Figure 2).

Quality of Evidence

The GRADE (Grading of Recommendations Assessment, Development and Evaluation) system was employed to appraise the quality of evidence from the total and subgroup analysis of the present review. It was initially classiﬁed as having high evidence in view of inclusion of all RCTs, but the evidence dropped to variant levels owing to significant publication bias, heterogeneity and risk of bias (Table 2). Hence, the quality of the evidence was low, which implies that the evidence from the pooled estimate effect was limited for the diverse analyses we had undertaken.

**Table 2:** Summary of evidence of the effect of vitamin C supplementation on the occurrence of PPROM and/or PROM CI: confidence interval; RCT: randomized controlled trial; OR: odds ratio; PROM: premature rupture of membranes; PPROM: preterm PROM Grades of evidence from the GRADE (Grading of Recommendations Assessment, Development and Evaluation) Working Group: High: high confidence in the match between the actual effect and estimated effect; Moderate: moderate confidence in the estimation of the effect. There is a possibility that the actual effect is far from the estimated effect; Low: limited confidence in the estimation of the effect. The actual effect may be far from the estimate; Very low: little confidence in the estimated effect. The true effect is most likely different from the estimate.

Parameters for metanalysis	Number of studies	Number of participants	Pooled effect size [95% CI]	Anticipated pooled effects	Certainty of the evidence (GRADE)
Vitamin C supplementation	9 RCTs [6-14]	Vitamin C: 8022, placebo: 8054	OR: 0.90 [0.55, 1.44]	The odds of the occurrence of PPROM and/or PROM in the intervention group were 0.90 lower. The overall effect estimate was not significant.	Very low
High-dose Vitamin C supplementation	5 RCTs [8-10,12,14]	Vitamin C: 7800, placebo: 7827	OR: 1.37 [0.92, 2.06]	The odds of the occurrence of PPROM and/or PROM in the intervention group were 1.37 higher. The overall effect estimate was not significant.	Very low
Low-dose Vitamin C supplementation	4 RCTs [6,7,11,13]	Vitamin C: 222, placebo: 227	OR: 0.28 [0.16, 0.48]	The odds of the occurrence of PPROM and/or PROM in the intervention group were 0.28 lower. The overall effect estimate was found to be significant.	Low
Vitamin C monotherapy supplementation	5 RCTs [6,7,11,13,14]	Vitamin C: 637, placebo: 645	OR: 0.41 [0.27, 0.63]	The odds of the occurrence of PPROM and/or PROM in the intervention group were 0.41 lower. The overall effect estimate was found to be significant.	Very low
Vitamin C combination supplementation	4 RCTs [8-10,12]	Vitamin C: 7385, placebo: 7409	OR: 1.52 [0.98, 2.35]	The odds of the occurrence of PPROM and/or PROM in the intervention group were 1.52 higher. The overall effect estimate was not significant.	Low
Vitamin C supplementation in high-risk women	2 RCTs [7,11]	Vitamin C: 115, placebo: 115	OR: 0.29 [0.15, 0.53]	The odds of the occurrence of PPROM and/or PROM in the intervention group were 0.29 lower. The overall effect estimate was found to be significant.	Very low

Description of the Included Trials

The nine eligible trials were undertaken in different countries, mostly middle- and high-income countries. The number of participants in the trials ranged from 62 to 9968 in different studies.

Patient Characteristics

Pregnant women in their second trimester of pregnancy were recruited for supplementation of vitamin C in all the studies. The control group consisting of antenatal women matched to gestational age was given placebo in most of the studies except for iron and folic acid tablets in one study [13]. The average age of patients ranged from 22 to 29 years in various studies. Most of the studies [6-8,12] recruited women with a singleton pregnancy while four studies [9-11,13,14] supplemented pregnant women irrespective of number of fetuses during the index pregnancy. None of the other studies mentioned the gravidity of pregnancy.

Study Characteristics

There were two studies [7,11] that analysed the favourable role of the micronutrient in preventing or averting the occurrence of PROM/PPROM in low-risk and high-risk pregnant women when supplemented in the second trimester of pregnancy. One study [13] supplemented iron and folic acid in both study and control groups. But we assume iron and folic acid were included in all trials since their supplementation in pregnancy is recommended for all women [16]. The dose of vitamin C varied in different studies. Some studies supplemented low doses of 100 mg [6,7,13] while one supplemented with intermediate doses of 500 mg [11] and rest used high doses of vitamin C of 1000 mg [8-10,12,14]. Studies supplementing vitamin E along with vitamin C used the same dose of 400 IU/day. Two studies [7,11] assessed the effect of the micronutrient in those who had a history of PPROM and PROM in their previous pregnancy while one study [9] studied its role in women having nonproteinuric chronic hypertension or a history of preeclampsia in the most recent pregnancy. Only three out of the nine studies included in the meta-analysis analysed the occurrence of PROM and/or PPROM as the primary outcome parameter [6,7,11]. Other studies interpreted the outcome as the secondary parameter with principal events being other pregnancy-related complications such as UTI [13], preeclampsia [5,9,10,14] and pregnancy-associated hypertension [8].

Synthesis of Results

**Figure 3:**
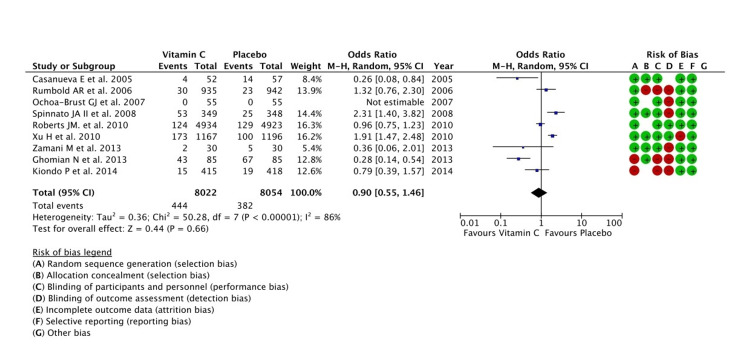
A forest plot comparison for the effectiveness of vitamin C in the prevention of PPROM and/or PROM PROM: premature rupture of membranes; PPROM: preterm PROM Refer to [6-14]

The effects of micronutrient supplementation on two selected outcomes related to PPROM and/or PROM are summarized in Table 2. A total of nine studies were included to evaluate the effect of vitamin C supplementation in the analysis. According to the Q-test, the true outcomes appeared to be heterogeneous (P < 0.0001, tau^2^ = 0.36, I^2^ = 86%). Hence, although the average outcome was estimated to be negative, in some studies the true outcome may in fact be positive. According to the Cook's distances, none of the studies could be considered to be overly influential. Nine trials with a total of 16,382 participants provided data on the occurrence of PROM and/or PPROM. In comparison to placebo, vitamin C supplementation was not shown to be significantly effective in preventing the occurrence of PPROM/PROM (OR = 0.90, 95% CI = 0.55, 1.46), P = 0.66. The trials, however, showed significant heterogeneity among them (tau^2^ = 0.36, I^2^ = 86%, P < 0.00001) (Figure 3).

Dose of Vitamin C

**Figure 4:**
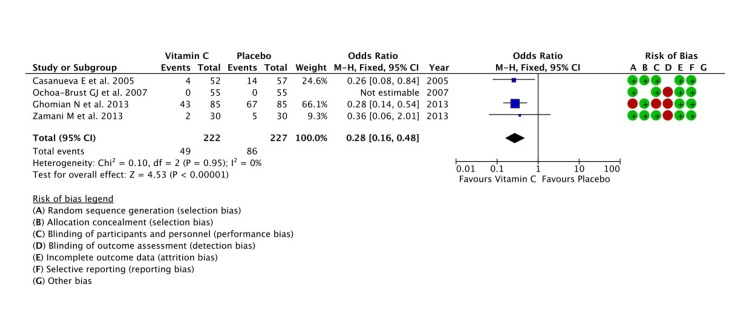
A forest plot comparison for the effectiveness of low-dose vitamin C (≤250 mg) in the prevention of PPROM and/or PROM PROM: premature rupture of membranes; PPROM: preterm PROM Refer to [6,7,11,13]

Vitamin C had been supplemented in varying doses in different trials ranging from 100 mg/day to 1000 mg/day. We assumed 250 mg/day and less than that as a low dose of the micronutrient. A low dose significantly decreased the occurrence of PPROM/PROM in pregnant women when compared to placebo (Z = 4.53; P < 0.00001). The studies reported little heterogeneity (tau^2^ = 0.00, I^2^ = 0%, P = 0.95) (Figure 4). When a high dose was considered, placebo exhibited better outcomes in preventing PPROM/PROM though the pooled effect estimate was not significant (Z = 1.34; P = 0.18).

Mode of Therapy

**Figure 5:**
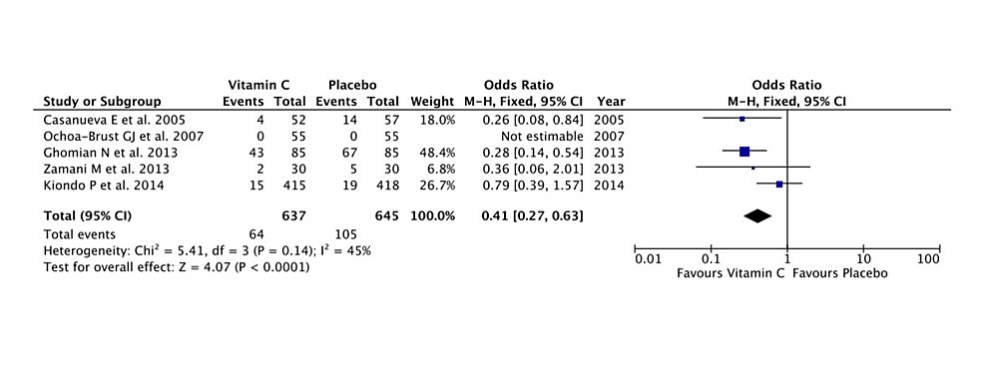
A forest plot comparison for the effectiveness of vitamin C monotherapy in the prevention of PPROM and/or PROM PROM: premature rupture of membranes; PPROM: preterm PROM Refer to [6,7,11,13,14]

Vitamin C had been supplemented as monotherapy as well as combination therapy. The pooled effect estimate showed monotherapy was beneficial in preventing PPROM/PROM and there was a significant difference in preventing the obstetric complication compared to placebo (Z = 2.82; P = 0.005) (Figure 5). Heterogeneity among studies was minimal (tau^2^ = 0.18, I^2^ = 45%, P = 0.14). However, placebo performed better when vitamin C was given in combination but was short of significance (Z = 1.57; P = 0.12). There was substantial statistical heterogeneity in the analysis.

PROM in the Previous Pregnancy

**Figure 6:**
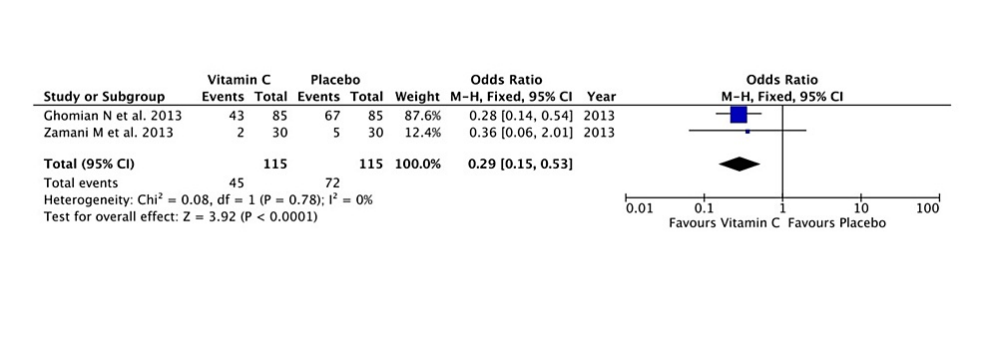
A forest plot comparison for the effectiveness of vitamin C in the prevention of PPROM and/or PROM in high-risk pregnant women PROM: premature rupture of membranes; PPROM: preterm PROM Refer to [7,11]

Women with a history of PROM in a previous pregnancy were shown to have significant beneficial effects with vitamin C supplementation (Z = 3.93; P < 0.0001) (Figure 6).

Publication Bias

**Figure 7:**
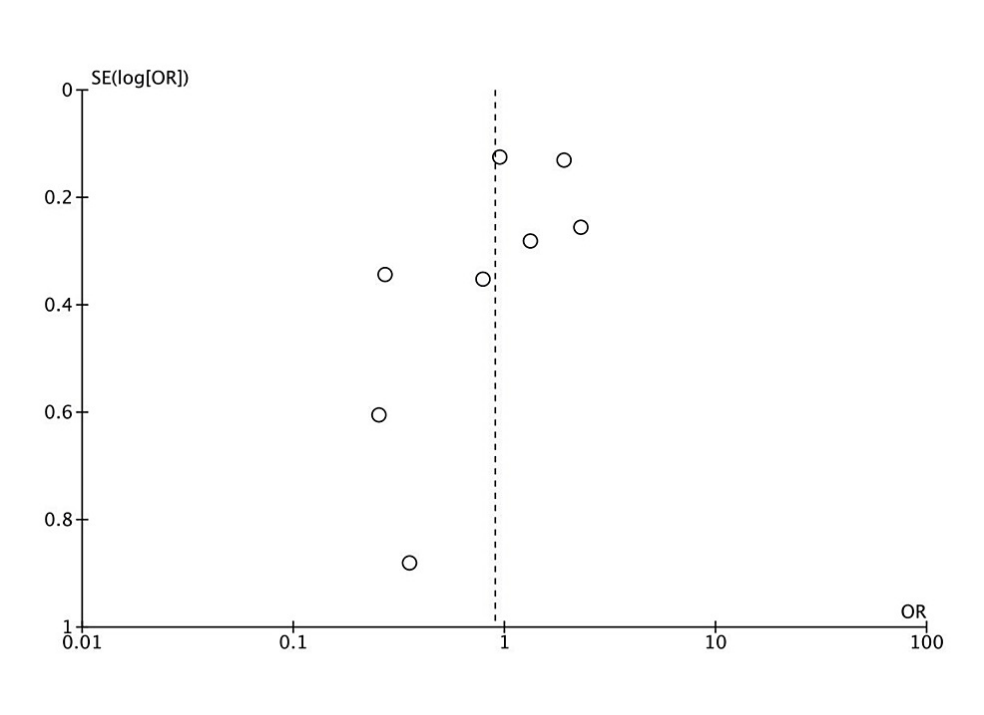
A funnel plot for estimates in the meta-analysis of vitamin C supplementation in the prevention of PPROM and/or PROM based on randomized controlled studies published before November 2022 PROM: premature rupture of membranes; PPROM: preterm PROM

The funnel plot constructed based on the data presented was visually and statistically significant for asymmetry as determined by a regression test for funnel plot asymmetry (Z = -2.133; P = 0.033) revealing small study effects (Figure 7).

Discussion

This review highlights the effect of vitamin C supplementation in preventing the occurrence of PPROM and/or PROM in pregnant women. The pooled estimate effect was not significant to draw a possible conclusion on the role of this micronutrient. The high between-trial variation reflected by significant heterogeneity and degrees of bias however limit our evidence of conclusion to recommend a definite practice of implementation. But the subgroup analysis revealed that the supplementation of low-dose vitamin C, vitamin C monotherapy and vitamin C in women with a high risk of PPROM/PROM has definite benefits in preventing the obstetric complication.

The role of vitamin C as an antioxidant is evident during periods of oxidative stress where it protects the host cells against the harmful effects of ROS thereby decreasing its levels during infections [17]. The micronutrient is also proved to be involved in the synthesis as well as the stabilization of collagen by cross-linking [18], and hence, a decreased vitamin C body reserve can cause disordered amniotic membrane synthesis with subsequent effects on the antioxidant activity and accelerated free radical-mediated tissue damage [19]. The association between the maternal vitamin C level and the incidence of PROM was first affirmed by Wideman et al. in 1964 [20]. However, in a systematic review and meta-analysis by Rumbold and Crowther, involving 10 trials and 16,825 participants, vitamin C was not found to decrease the risk of PPROM significantly (RR = 0.98, 95% CI: 0.7, 1.36) [5]. Another such meta-analysis by Conde-Agudelo et al. reported significant increase in the risk of PROM and a non-significant increase in the risk of PPROM after the micronutrient supplementation [21]. The similar findings of our study certainly contrapose the pathophysiologic role of vitamin C in maintaining collagen integrity. This, however, prompted us to look for effects following changes in dose, mode of therapy and supplementation in high-risk women.

The studies included in our review supplemented vitamin C in doses varying from 100 mg/day to 1000 mg/day. The low-dose regimen was found to lower the incidence of PPROM/PROM significantly. The individual effect sizes were significant in all the trials except in the study by Zamani et al. [11] where its 95% confidence interval crossed the line of no effect. The ramifications of the diverse dosing regimens invite deeper speculations into the pathophysiology of the obstetric complication [11]. Several studies provided evidence that vitamin C induces a pro-apoptotic impact on diverse human cells [22,23] along with the decomposition of lipid hydroperoxides [24]. Even the exacerbation of hydrogen peroxide-induced apoptosis was reported with vitamin C pre-incubation in an amnion-derived WISH cell model [25]. However, Mühlhöfer et al. deduced that high intravenous doses of vitamin C may not produce pro-oxidant effects in vivo [26]. But it was a crossover study design where only six healthy individuals were enrolled. A sampling bias in view of an unrecognized prior serum vitamin C status of the individuals was a possibility that cannot be disregarded. Hence, the probable speculation of Mercer et al. that high doses of vitamin C may induce the degradation and apoptosis of vitamin C appears appropriate that supports our pooled analytic results [27].

According to Gupta et al., women with PPROM had significantly lower levels of serum vitamin C when compared to BMI- and gestational age-matched controls [28]. Another study also discovered an association between PPROM and a low dietary vitamin C intake [4]. These studies illustrate a different assertion of the obstetric phenomenon in relation to dosing of the micronutrient. The questions arise as to the variation in the response with regard to the serum vitamin C status of an individual. Will the response and effect deviate with the sufficient or deficient status of the pregnant women? None of the trials included in the meta-analysis determined the plasma levels prior to supplementation. Hence, the dosing protocols may need revision with regard to the status of body stores. The presumption of a high dose of vitamin C having a positive correlation with the occurrence of PPROM/PROM therefore needs further exploration before we undermine its effects. In view of the high heterogeneity (I^2 ^= 81%) and a non-significant pooled effect along with a very low grade, recommendations against the high-dosing protocol will need evidence with robust prospective trials in deficient and sufficient individuals to validate its disapproval.

Vitamin C when supplemented alone without vitamin E decreased the incidence of PPROM/PROM in pregnant women significantly (P < 0.0001). The pooled effect size favoured placebo therapy when vitamin C was administered in combination with vitamin E; however, it was just short of significance (P = 0.06). Vitamin E cannot function alone and works in synergy with vitamin C, vitamin B3, selenium and glutathione. An optimum effect of vitamin E will be divulged with other nutrients in a balanced amount either provided by diet or supplementation [29]. The mechanism driving the role of vitamin E in the event of deficient or sufficient status of the other micronutrients needs to be elucidated and hence any assumption and inference from findings as mentioned above need to be drawn with caution. The interplay between the effects of the micronutrients as well as the evaluation of the independent effects of the micronutrients needs research and exploration.

Traber and Atkinson [30] in their traditional review inferred that vitamin E supplementation has no impact on the markers of oxidative stress in individuals not under any stress. They proposed that the different signalling pathways assumed to be regulated by α-tocopherol depend on the status of oxidative stress of the cell or tissue under consideration. When considering the occurrence of PPROM/PROM, the body tissues are under stress, which is assumed to kickstart the onset of labour. In the light of our finding from pooled results that vitamin C supplementation has a definite benefit in women with a history of PPROM/PROM in a previous pregnancy, such proposition by the authors [30] seems pertinent.

The multiple subgroup analysis giving us a clear review of the effect variation pertaining to changes in the dose of vitamin C, mode of therapy and supplementation in those with a history of PPROM is the strength of our study. None of the earlier analyses investigated into the depths of these multiple pooled effects, hence precluding the former perception that vitamin C supplementation has got no role in the prevention of PPROM/PROM. The comprehensive analysis of the effect of vitamin C as antenatal therapy from the second trimester was done adhering strictly to PRISMA guidelines. Our review has some limitations too. The subgroup analyses were undertaken with a limited number of studies. The number of participants were less too.

## Conclusions

In conclusion, vitamin C supplementation in pregnancy should be an individualised protocol customised to each pregnant woman taking into account her prior obstetric history and probable serum vitamin C levels. The dose should be modified too with regard to her clinical status and where possible, a low dose, preferably 100 mg/day, should be started. Those with a history of PPROM/PROM should be offered the benefit of the micronutrient supplementation.
